# Road traffic can be predicted by machine learning equally effectively as by complex microscopic model

**DOI:** 10.1038/s41598-023-41902-y

**Published:** 2023-09-04

**Authors:** Andrzej Sroczyński, Andrzej Czyżewski

**Affiliations:** Multimedia Systems Department, Faculty of Electronics, Telecommunication and Informatics, Gdansk University of Technology, 80-233 Gdańsk, Poland

**Keywords:** Information technology, Computer science

## Abstract

Since high-quality real data acquired from selected road sections are not always available, a traffic control solution can use data from software traffic simulators working offline. The results show that in contrast to microscopic traffic simulation, the algorithms employing neural networks can work in real-time, so they can be used, among others, to determine the speed displayed on variable message road signs. This paper describes an experiment to develop and test machine learning models, i.e., long short-term memory, gated recurrent unit recurrent networks, and stacked autoencoder networks. It compares their effectiveness with traffic prediction results generated using a widely recognized traffic simulator that analyzes traffic at the level of individual vehicles.

## Introduction

Over the past decade, intelligent transportation systems (ITS) have become an essential research object and continue developing rapidly. ITS combines information, communications, and transportation technology to optimize traffic management, improve road safety, increase the efficiency of transportation networks, and improve public transportation management. An important factor affecting the quality of ITS operations, especially in the context of traffic control, is traffic volume information, both long-term and short-term. Individual and business road users particularly desire the latter. Because of the stochastic and nonlinear nature of the traffic phenomenon, accurately predicting it in real time has been and remains a difficult task^1^. The methods used so far mainly rely on linear and shallow machine learning models to predict traffic flow. These methods cannot correctly reflect the non-linearity and probabilistic characteristics of the phenomena, although they can respond to seasonal changes^2,3^. In recent years, there have been successful alternative attempts at traffic prediction based on deep machine learning methods^1,4–6^, and methods based on combining different models, such as ARIMA and LSTM^7^, Kalman filters, and ARIMA^8^. More complex strategies are also being used to improve prediction accuracy, such as the combined use of the light gradient boosting machine (LightGBM) and the gated recurrent unit (GRU) with the SARIMA-GRU prediction model that takes into account the periodicity of traffic phenomena^9^. Despite advances in AI and machine learning, the importance of traditional statistical methods should not be diminished^3^. Among those often found in publications are GRU time series prediction methods, LSTM NNs^10^, and stacked autoencoders (SAE)^1^. In cases of large, complex motion models, the standard practice is to combine spline-recurrent neural networks with a motion graph^11,12^. This method allows the influence of traffic on neighboring roads to be considered. It is particularly effective when modeling large, branching road networks, such as in large metropolitan areas^11,13–16^. The surprisingly rapid development of machine learning methods has impressively increased the number of possible types and configurations of neural networks, but so far, this progress has not extended to the problem of optimization understood both in terms of choosing the most suitable model and its architecture (e.g., the number of hidden layers is still chosen arbitrarily) and the selection of its internal parameters^10^. This problem is general and does not apply only to traffic prediction. Due to the traffic phenomenon locally serial nature, the GRU, LSTM, and stacked autoencoders (SAE) neural network models mentioned above are often adopted for their analysis^10^. It goes without saying that to run any machine learning model, data collected in a real-world environment is necessary. An excellent and frequently used source of traffic data is the Caltrans Performance Measurement System (PeMS)^17^. Software traffic simulators can be an auxiliary data source when real data is unavailable or insufficient, such as not covering the required time range. In this article, we present the results of an experiment testing LSTMs, GRUs, and SEAs models trained on real data with data acquired using the Vissim^18^ simulator based on the traffic model ruling the movement of vehicles proposed by R. Wiedemann at Karlsruhe University. The definition of the problem to be solved in the presented research is as follows:

1. A large real-world traffic data trains and validates the machine learning model,
2. We would also like to assess prediction accuracy using data from other sources, such as computer simulations,
3. Developed neural network models are applicable to determine the speed to be displayed on variable message road signs, for example, of the kind we have constructed previously^19^.

The remainder of this attribution is organized as follows: in “Related work” section contains a brief literature review, in “Traffic simulation methods” section describes popular traffic simulation methods, in “Dataset and tests” section describes the actual data used in the experiment and a description of the investigation, and in “Results” section presents the results obtained. Finally, the results and conclusion are discussed in “Discussion” and “Conclusions”, respectively.

## Related work

The extensive literature on the application and use of various machine-learning techniques for traffic prediction continues to increase. However, given the number of available publications, this review can only provide a cursory glimpse into the current state of the vast literature on the subject.

Over the past decade, many different method proposals have been made for traffic prediction. The proposed methods were initially based on the autoregressive integrated moving average model (ARIMA). However, it turned out that the ARIMA model, which is based on linear relationships, cannot analyze the nonlinear and time-varying relationships that are an inherent feature of traffic^5^. Furthermore, the ongoing development of methods related to deep learning in recent years has led to the emergence of new proposals, in particular SAE, DNN, DBN, LSTM, or CNN-LSTM, as well as methods that are combinations of them.

One of the many examples of this approach is a publication^5^, whose authors propose a combination of CNN and LSTM (Conv-LSTM) methods for extracting spatial–temporal properties and a two-kernel LSTM (Bi-LSTM) model for extracting periodic traffic properties. According to the authors, the proposed method gives better results than the other methods mentioned in the comparison. The ARIMA and SAE methods performed particularly unfavorably. It is also worth noting that the quality of the prediction of the presented solution was greatly affected by the addition of the Bi-LSTM module. 

A slightly different approach to the problem of spatial traffic analysis shows Li et al.^20^. The authors suggest modeling traffic as a diffusion process on a directed graph. They propose the diffusion convolutional recurrent neural network (DCRNN) for traffic prediction. This framework represents a holistic approach and considers spatial and temporal traffic conditions. According to the authors, the experiments showed the clear superiority of DCRNN over other current methods.

The LSTM method already has an established positive reputation. Mou et al.^21^ present the results of a study on the influence of time variation on the results of LSTM prediction. The proposed improvement considers the mentioned variability and introduces the T-LSTM method. The authors also use this method to solve the problem of missing data. The results obtained employing experiments confirm the high efficiency of this method proposed in the literature.

A new paper, published in April 2023, proposes a hybrid STFSA-CNN-GRU model for short-term traffic speed prediction^22^. This model eliminates the limitations of single architecture and provides good accuracy and stability for multiple predictions.

The difficulties of traffic prediction were highlighted in a review article from June 2022, which is also relatively recent^23^. The article authors cite the complexity of spatial–temporal relationships, the dynamics of temporal dependencies, and the influence of external factors such as weather conditions and traffic events as challenges. According to its authors, the publication is the first comprehensive survey of techniques currently used for traffic prediction based on deep machine learning. The review shows that in terms of spatial modeling, the dominant methods are CNNs and those based on Chebyshev polynomials. On the other hand, temporal parameter modeling most often uses RNNs, including variants of these networks, namely LSTM^24,25^ or GRU. Such a structure is repeated in all areas of traffic prediction, i.e., flow prediction, vehicle speed, demand, travel time, and occupancy. This work gives a good indication of the complexity of the modeling techniques currently used to study traffic, a phenomenon, as it turns out, that is also very complex.

Ghosh et al.^26^ propose a one-dimensional structural time series model (STM) used to develop an economical and computationally simple multidimensional time model algorithm. In the STM methodology, various components of a time series dataset, such as trend, seasonality, cyclical and calendar components, can be modeled separately. The results indicate that the proposed forecasting algorithm is a practical approach for real-time traffic flow prediction at multiple intersections in an urban transportation network.

Deep learning techniques are also used in solutions for tracing and reconstructing vehicle traffic trajectories. Fei et al.^27^ use CNN techniques to analyze images from multiple cameras to identify and locate objects, including pedestrians.

A solution based on deep neural networks (DNNs) is discussed in a publication^28^. The authors propose a six-layer DNN for long-term traffic prediction based on historical data and a set of coefficients that define the context of traffic conditions, such as day of the week, weather, and season. The primary assumption of the presented method is a strong correlation of vehicle stream flow with the starting and ending time points of a short observation period, along with contextual factors. Making such an assumption makes it possible to extract the relationship between traffic flow values in a given time interval from historical data and the combination of contextual factors.

An essential aspect of traffic analysis is the prediction of accident risk. Trirat et al.^29^ propose multi-view graph convolutional networks for traffic accident risk prediction (MG-TAR). The presented approach attempts to solve two non-trivial problems: understanding and deriving dangerous driving statistics based on rule-based classification and modeling simultaneous static and dynamic graphs representing traffic conditions. An exciting application of deep convolutional neural networks (CNNs) is the method presented in the publication^30^. Its authors propose a method for analyzing a large-scale transportation network based on generating, using CNNs, a graphical image containing traffic information in the analyzed transportation network.

An interesting approach to traffic prediction based on incomplete data is using neural networks with an architecture that uses graph Markov processes–graph Markov network (GMN)^31^. An extension of this method by adding spectral graph splicing operations is the second method proposed by the authors, called the 'spectral graph Markov network' (SGMN). According to the publication authors, both methods, GMN and SGMN, achieve good prediction results in accuracy and performance. Among the most recent publications, a very interesting one seems to be^32^, whose authors present a new approach to this problem, i.e., traffic prediction for incomplete data. Also worth mentioning is publication^33^, which provides a comprehensive review and comparison of traffic prediction methods used. Very interesting results were obtained by Abduljabbar^34^ using two-way LSTM (BiLSTM) models. The developed BiLSTM short-term traffic forecasting models were evaluated using data from a calibrated microsimulation model.

An interesting idea is the use of deep neural network methods for 'backward prediction', i.e., to reconstruct the conditions existing before the collision employing damage and deformation analysis of the vehicles involved in the crash. Promising research results were presented by Chen et al.^35^. Experiments carried out using the three-neural network (3NN) algorithm on synthetic data confirm the considerable potential of the concept adopted.

A slightly different approach was proposed by Xie et al.^36^ They used variational bayesian learning theory and a feed-forward neural network architecture for ‘inverse prediction’. As in^35^, experiments were carried out based on synthetic data, but the results are promising. The development of methods and algorithms that allow the reconstruction of pre-collision conditions based on the results of a collision is significant in forensic accident analysis for vehicle designers and—most notably in the context of the main topic of this article—can assist ITS in the accident prevention process by anticipating events based on the pre-collision conditions memorized by AI algorithms.

## Traffic simulation methods

Several traffic simulation methods differ in the level of detail, the complexity of modeling, range, and accuracy of the simulation. Among the most commonly used are:

- Microscopic traffic simulation—this method allows simulating traffic at the level of individual vehicles, taking into account their movement, maneuvers, acceleration, braking, lane changes, etc. This type of simulation is applied to a limited area of the designed transportation network and is often used to assess the effects of changes in road infrastructure.
- Macroscopic traffic simulation—this method is applied to large areas of the transportation network, allowing traffic to be simulated at the level of vehicle flow and congestion, considering only the overall traffic and capacity of the system. This type of simulation is often used to analyze the ability of transportation systems and assess the effects of changes in road geometry.
- Mesoscopic traffic simulation—this method combines microscopic and macroscopic traffic simulation, allowing simultaneous consideration of the details of individual vehicle movement and the overall flow and congestion of the road system. In the mesoscopic simulation, models consider individual system elements and group elements. It means mesoscopic simulation allows behavior analysis at individual and collective levels but with less accuracy than in microscopic simulation.
- The phenomena occurring in traffic and their probabilistic nature make it impossible to map them with sufficient accuracy using analytical methods. Hence, it is now standard practice to use computer simulations. VISSIM, AIMSUN, PARAMICS, TRANSIMS, and SUMO are among the most well-known and popular traffic simulation programs. The latest versions of some of these programs use machine learning and artificial intelligence techniques.

## Dataset and tests

Two data sets were used for the experiments:

1. Actual measurement data provided by the California Department of Transportation (CalTrans) on the Caltrans Performance Measurement System (PeMS) website^17^. The use of PeMS data requires some caution, as it is quite common for individual measurement stations or circuits to fail to provide reliable measurement data due to damage or shutdowns. The technical condition of the measuring equipment and the evaluation of the reliability of the data can be checked on the PeMS website.
2. The synthesized data set was obtained using Vissim microscopic simulation software^37^.

For the training and validation of the machine learning models, data of May 2022 was selected and divided into a learning set of 4377 records, containing traffic volume data from May 1st to 16th, and a validation set of 1095 records, containing data from May 16th to 19th. In both cases, data were aggregated at a 5-min interval and came from station 772903 (I210-E).

The test data was generated using the Vissim simulator, calibrated with PeMS data from May 1st to 7th.

### Description of experiments

The methodology used in the first phase of experiments was initially inspired by publications by Lv et al.^1^ and Fu et al.^10^. Tests were performed based on three machine learning models: LSTM, GRU, and SAE. A formal description of these algorithms will not be included here, as they are repeated many times in the cited literature and many tutorials available on the Internet.

**Figure 1:**
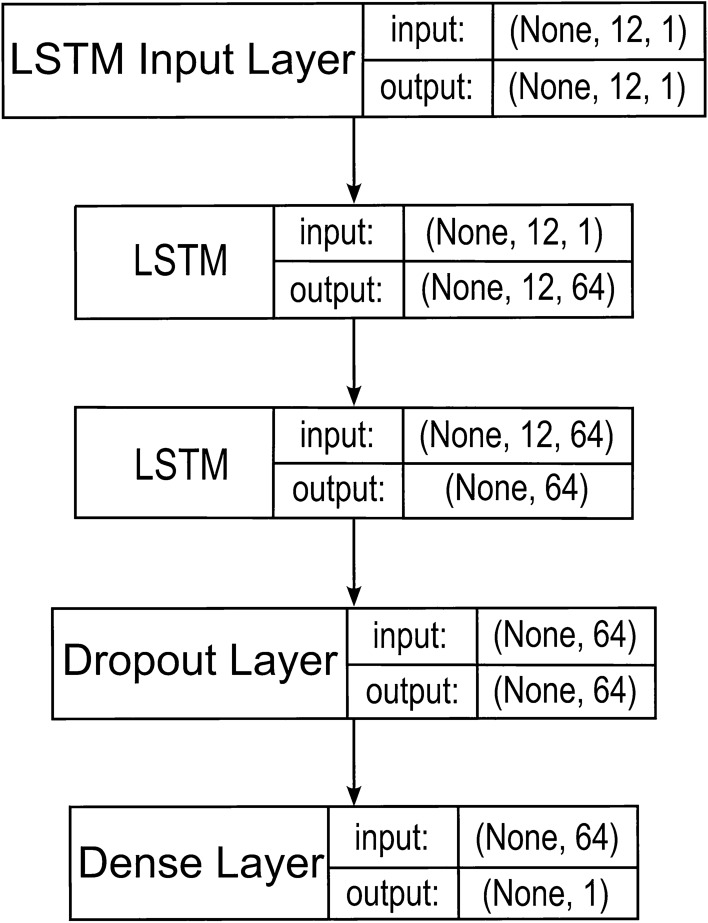
LSTM model.

The adopted LSTM model in a three-layer configuration is shown in Fig. 1.

Four models containing one to four layers of LSTM were made for further testing.

**Figure 2:**
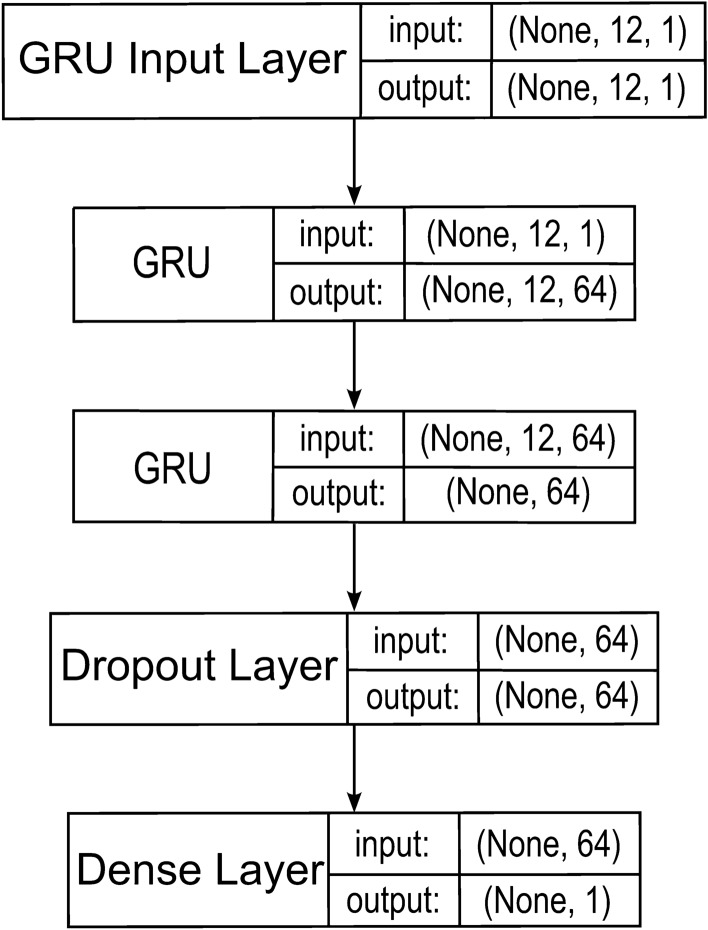
GRU model.

The adopted GRU model in a three-layer configuration is shown in Fig. 2.

As with the LSTM, four models containing one to four GRU layers were made.

**Figure 3:**
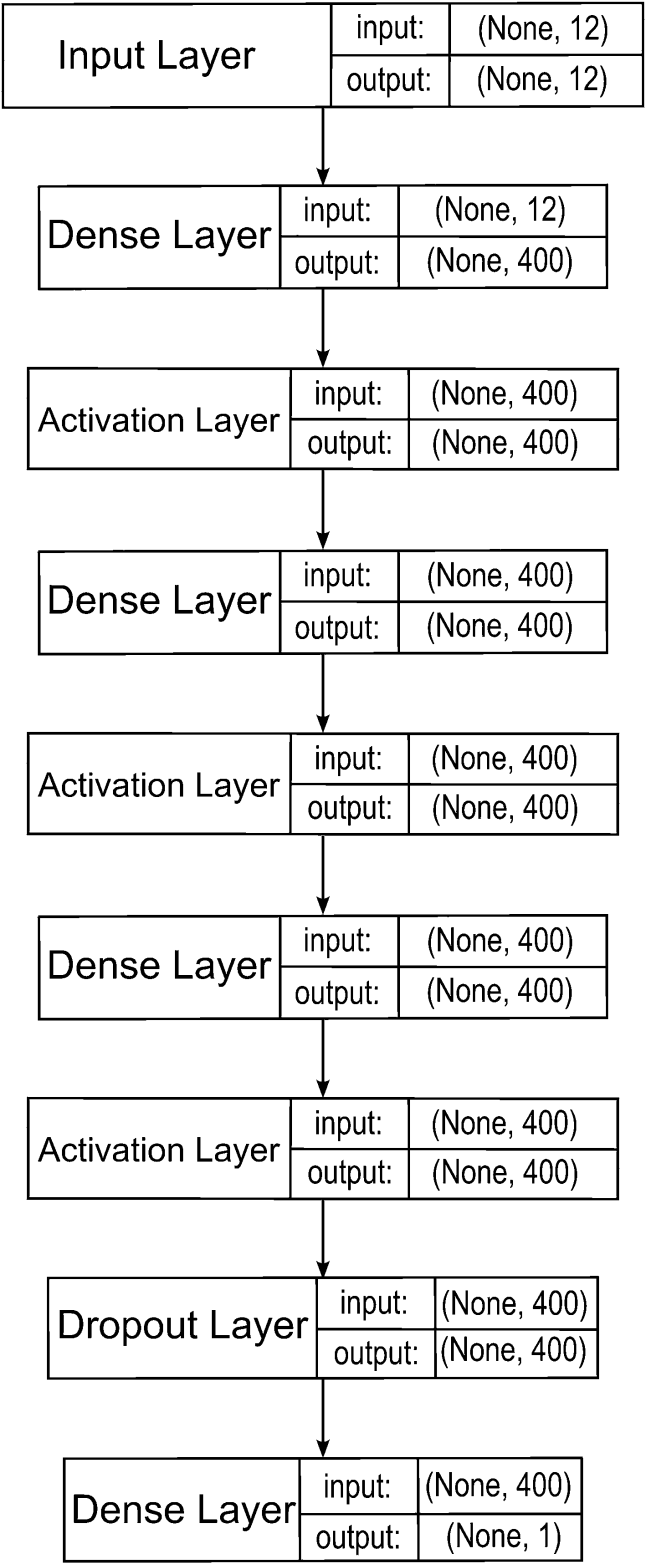
SAE model.

The SAE model used is shown in Fig. 3.

The SAE model was tested only in the three-layer configuration shown above. The reason for this was that pilot tests showed that for stacked autoencoders the average percentage error between the predicted and actual value for the validation set tends to be higher than for GRU and LSTM models. For this reason, we paid more attention to testing models based on recurrent networks rather than autoencoders. Like its predecessors, it was tested using synthetic data derived from the Vissim simulator after being trained on real data. The following metrics were used to evaluate the models: explained_variance_score, mean absolute percent error (MAPE), mean absolute error (MAE), mean squared error (MSE), rooted mean squared error (RMSE), and determination coefficient R2.

These metrics can be expressed as follows:

1 $$Explained\, variance\left(y, {\hat{\text{y}}} \right)=1- \frac{Var(y-  {\hat{\text{y}}} )}{Var(y)}$$

where *y*—actual values, $\hat{y}$*—*predicted values, *Var*(*y* − $\hat{y}$)—variance of prediction errors, Var(y)—actual variance values

2 $$MAPE\left(y, {\hat{\text{y}}} \right)=\frac{1}{n}\sum_{i=1}^{n}\left|\frac{{y}_{i}-{ {\hat{\text{y}}} }_{i}}{{y}_{i}}\right|$$

3 $$MAE\left(y, {\hat{\text{y}}} \right)=\frac{1}{n}\sum_{i=1}^{n}\left|{y}_{i}-{ {\hat{\text{y}}} }_{i}\right|$$

4 $$MSE\left(y, {\hat{\text{y}}} \right)=\frac{1}{n}\sum_{i=1}^{n}{\left({y}_{i}-{ {\hat{\text{y}}} }_{i}\right)}^{2}$$

5 $$RMSE\left(y, {\hat{\text{y}}} \right)=\sqrt{\frac{1}{n}\sum_{i=1}^{n}{({y}_{i}-{ {\hat{\text{y}}} }_{i})}^{2}}$$

7 $$R2=\frac{\sum_{i=1}^{n}{\left({ {\hat{\text{y}}} }_{i}-\overline{y }\right)}^{2}}{\sum_{i=1}^{n}{\left({y}_{i}-\overline{y }\right)}^{2}}$$

8 $$\overline{y }=\frac{1}{n}\sum_{i=1}^{n}{y}_{i}$$

All calculations were performed on a computer with a six-core Intel Core i7-6800k processor @ 3.40 GHz, 64 GB RAM, with an NVIDIA GeForGTX 1070 graphics card, running MS Windows 10 Pro.

**Figure 4:**
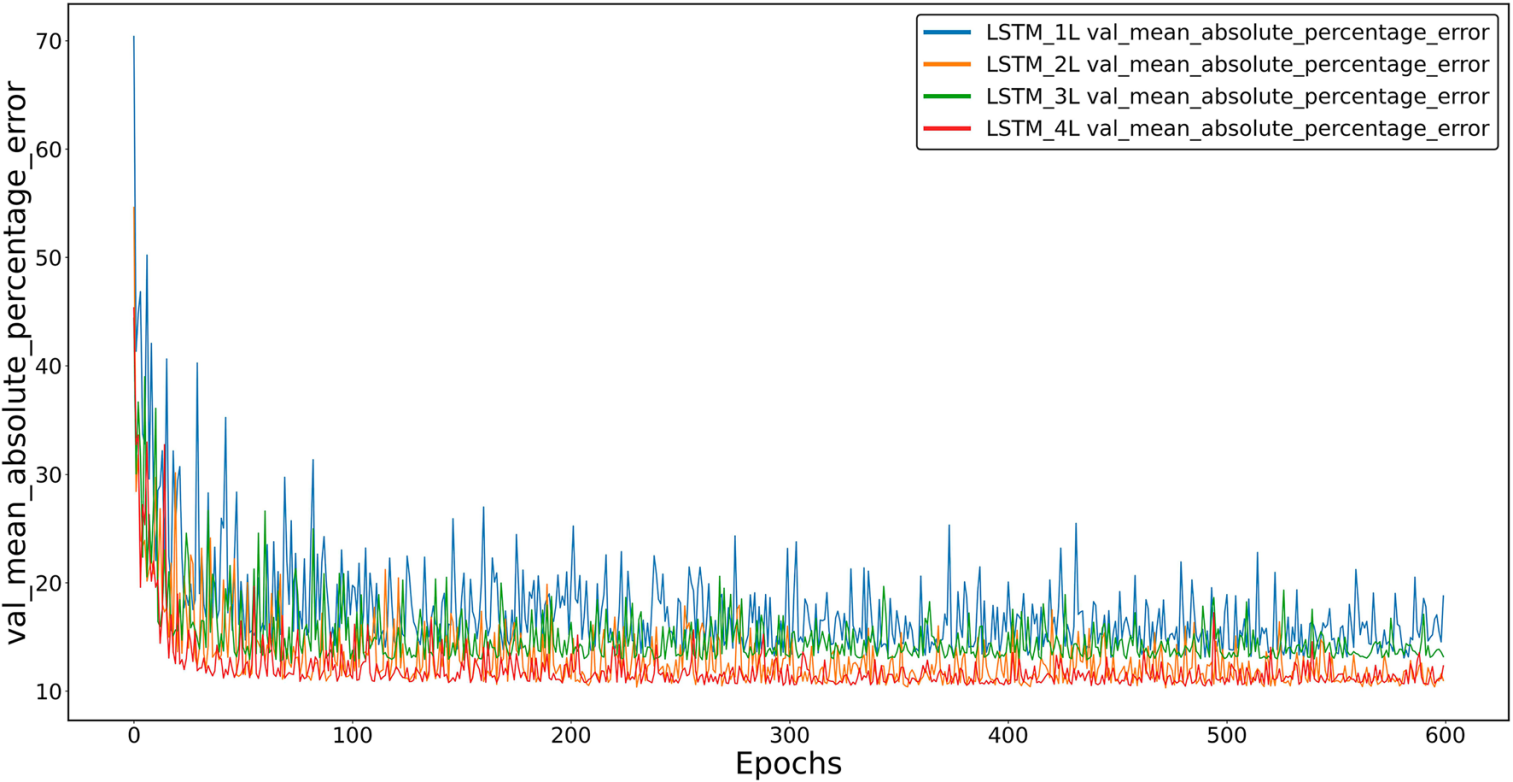
Average percentage error between the predicted and actual values for the validation set of four LSTM models: one-, two-, three- and four-layer.

Figure 4 shows the average percentage error (mean_absolute_percentage_error) as a function of the number of learning loops for the validation set and four LSTM models, differing in the number of inner layers.

It can be seen that learning loss is not a linear function of the number of layers and that it is the smallest in cases where the number is an even number.

**Figure 5:**
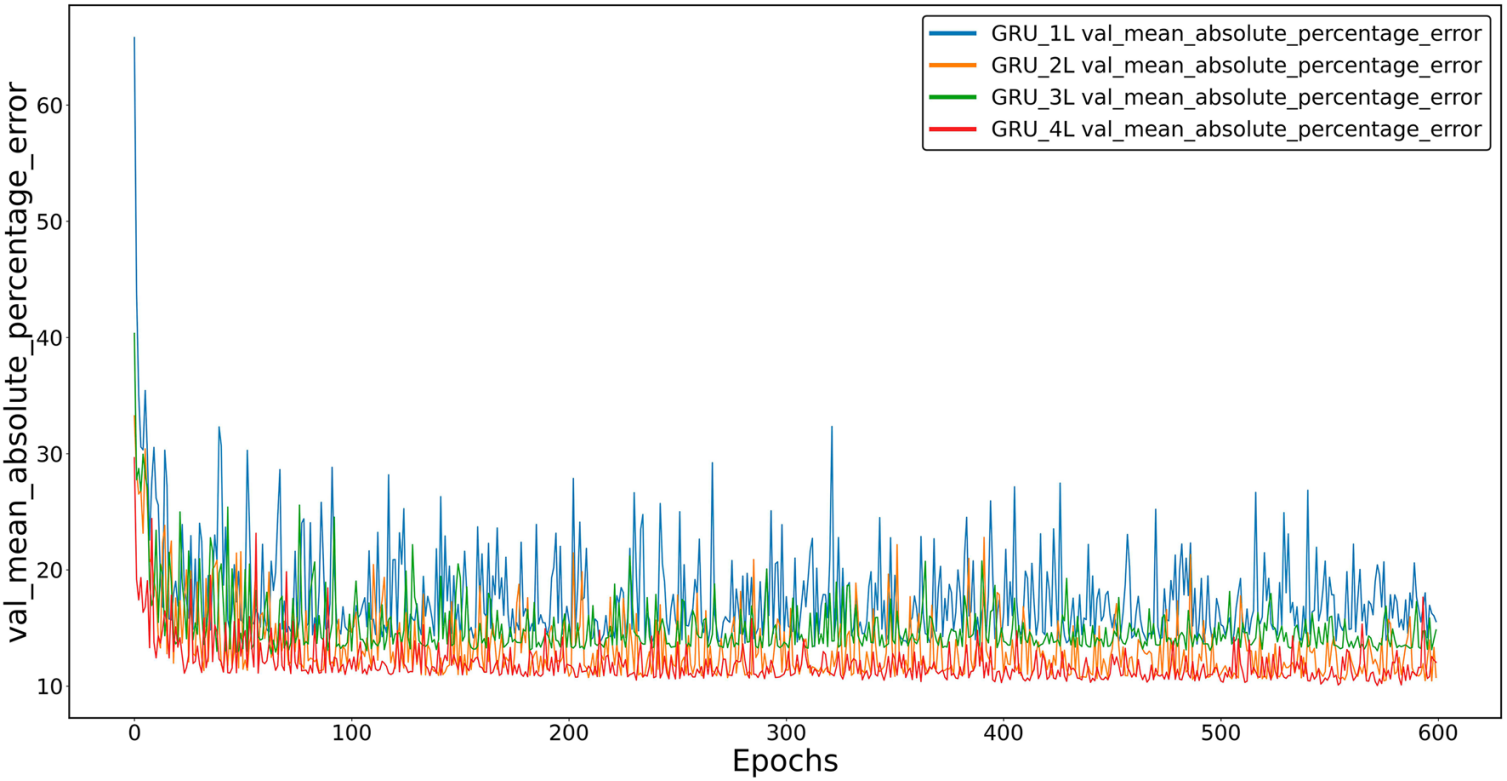
Average percentage error between the predicted and actual values for the validation set of four GRU models: one-, two-, three- and four-layer.

Similarly, Fig. 5 shows the average percentage error (Mean_absolute_percentage_error) as a function of the number of learning loops for a validation set and four GRU models, differing in the number of inner layers.

**Figure 6:**
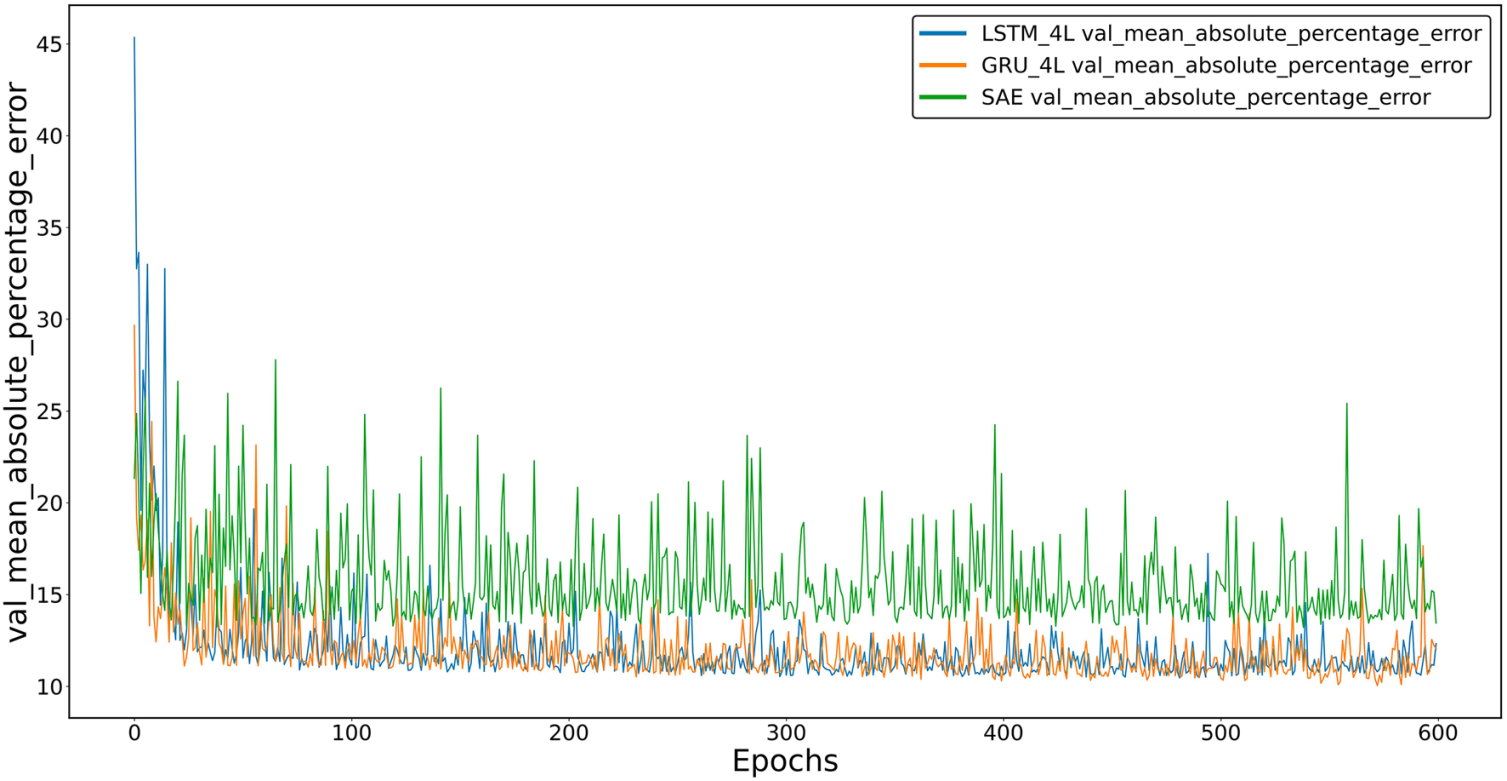
Average percentage error between the predicted and actual value for the validation set of the SAE model and the four-layer GRU and LSTM models.

As with LSTM, models with an even number of inner layers show the lowest learning losses. However, for all GRU models, the losses appear higher than for LSTM. A comparison of losses for the three models tested is shown in Fig. 6.

While the learning losses of the GRU and LSTM models are comparable, with the LSTM indicated as the slightly more accurate model, the learning losses of the SAE model are significantly higher.

### Vissim program calibration

**Figure 7:**
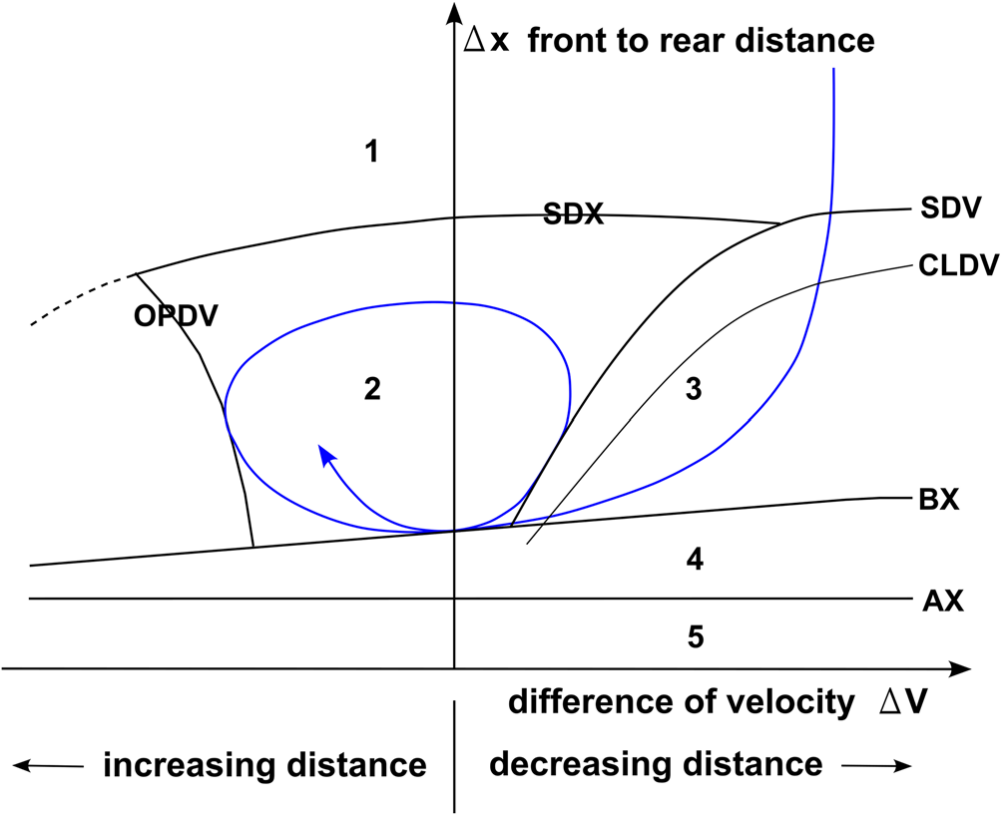
Car following model (according to Wiedemann, 1974). **1**: "Unregulated behavior” state (no reaction); **2**: Following state; **3**: Approaching state; **4**: Braking state; **5**: Collision state; AX: stationary distance; BX: min the following distance; CLDV: perception threshold (near): speed higher than leader; SDV: perception threshold (far): speed higher than the leader; OPDV: perception threshold: speed lower than the leader; SDX: perception threshold: free acceleration.

The Vissim software provides two psychophysical models to describe driver behavior: Wiedemann 74^38^ and Wiedemann 99^39,40^. The general structure of both models in terms of car-following mode is similar and shown in Fig. 7. The Wiedemann 99 model is more sophisticated and has many more parameters that need to be set (calibrated). This model is designed for traffic analysis at higher speeds (highways, expressways). Simulations were carried out based on the Wiedemann 99 model.

Following the recommendations presented in the literature^41^, the following values of calibration parameters were adopted (parameter names according to Vissim):

**Table:** 

CC0 (standstill distance):	1.5 m
CC1 (headway time):	0.9 s
CC2 ('following' distance oscillation):	4 m
CC3 (the threshold for entering ‘following’):	− 8
CC4 (negative 'following' threshold):	− 0.35
CC5 (positive 'following' threshold):	0.35
CC6 (speed dependency of oscillation):	11.44
CC7 (oscillation acceleration):	0.25 m/s^2^
CC8 (standstill acceleration):	3.5 m/s^2^
CC9 (acceleration at 80 km/h):	1.5 m/s^2^

It was assumed that only passenger cars (90.1%) and trucks (9.9%) participate in the traffic stream and that the permitted speeds, according to US regulations, are 70 mph and 55 mph, or 112.6 km/h and 88.5 km/h, respectively. Traffic volume distribution was defined based on PeMS data from May 1st to May 7th.

## Results

Figures 4, 5, 6, 7, 8, 9, 10 and 11 illustrate the results obtained from the experiments, i.e., the prediction results obtained with the models described above. All of the following traffic volume graphs have been normalized to facilitate the evaluation of the results.

**Figure 8:**
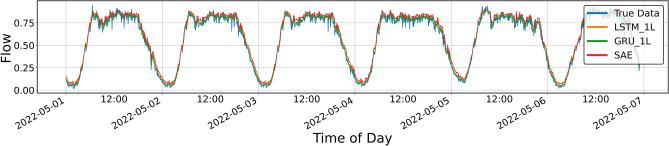
Prediction result for SAE and 1-layer LSTM and GRU models, prediction period of 5 days.

**Figure 9:**
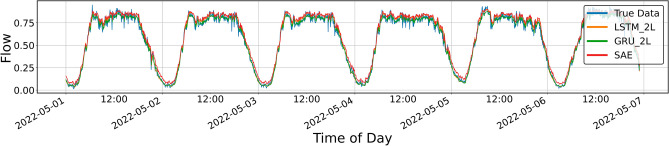
Prediction result for SAE and 2-layer LSTM and GRU models, prediction period of 5 days.

**Figure 10:**
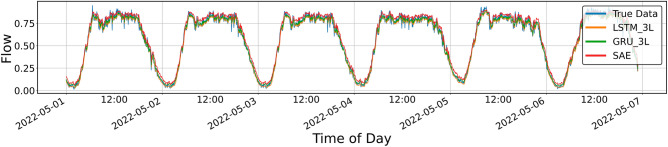
Prediction result for SAE and 3-layer LSTM and GRU models, prediction period—5 days.

**Figure 11:**
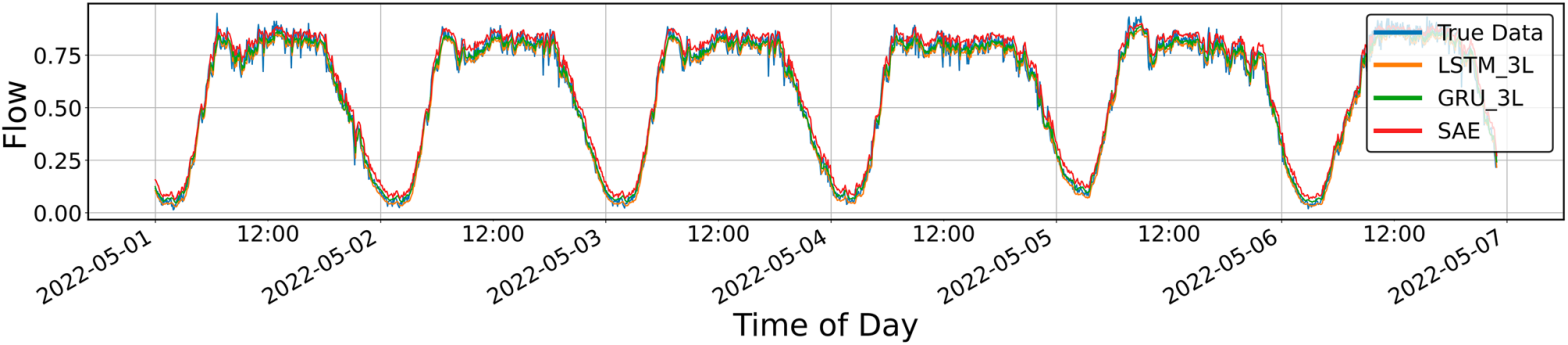
Prediction result for SAE and 4-layer LSTM and GRU models, prediction period of 5 days.

Figure 8 shows the prediction results for the single-layer LSTM and GRU models and the SAE model. As mentioned, the SAE model was tested only in the three-layer configuration described above. Therefore, in the remainder of this article, only the name SAE will be used, without mentioning the details of its architecture.

The resulting graph does not reveal significant differences between the models, but it can be seen that the upper sections of the curves do not coincide with the line drawn by the actual data. The numerical data of the parameters included in Table 1 equally do not show significant differences, except for *MAPE* (mean absolute percentage error—loss function), which for the SAE model is clearly more significant than for the other models.

**Table 1:** Error metrics for the prediction shown in Fig. 8.

	LSTM	GRU	SAE
Explained_variance_score	0.981526	0.982227	0.982012
MAPE	0.079151	0.081650	0.090258
Mae	0.029685	0.031415	0.029561
MSE	0.001531	0.001633	0.001487
RMSE	0.039128	0.040408	0.038562
R2	0.981221	0.979973	0.981761

Figure 9 shows the prediction results for the two-layer LSTM and GRU models and the SAE model. It can be seen that the graph corresponding to the GRU model is clearly below the LSTM and SAE lines. The *MAPE* numerical values shown in Table 2 confirm this observation: the error for GRU is more significant.

**Table 2:** Error metrics for the prediction shown in Fig. 9.

	LSTM	GRU	SAE
Explained_variance_score	0.982144	0.978448	0.981211
MAPE	0.086983	0.109168	0.079977
MAE	0.029304	0.035923	0.030026
MSE	0.001461	0.002053	0.001532
RMSE	0.038217	0.045307	0.039146
R2	0.982086	0.974822	0.981204

Figure 10 shows the prediction results for SAE and the three-layer LSTM and GRU models. In this case, no significant differences can be seen between the predictions, and the numerical values shown in Table 3 are similar, although the *MAPE* value for GRU is the largest.

**Table 3:** Error metrics for the prediction shown in Fig. 10.

	LSTM	GRU	SAE
Explained_variance_score	0.982213	0.982460	0.981234
MAPE	0.084427	0.089500	0.082789
MAE	0.031964	0.029042	0.030540
MSE	0.001666	0.001433	0.001550
RMSE	0.040818	0.037861	0.039366
R2	0.979565	0.982418	0.980993

Figure 11 shows the prediction results for the SAE model and the four-layer LSTM and GRU models. As in previous cases, only the *MAPE* shows more significant differences, while the other parameters are very similar. For GRU, the *MAPE* takes on a value similar to that for the two-layer model, clearly more significant than that shown for LSTM and SAE (Table 4).

**Table 4:** Error metrics for the prediction shown in Fig. 11.

	LSTM	GRU	SAE
Explained_variance_score	0.979126	0.980331	0.980191
MAPE	0.094460	0.104544	0.086037
MAE	0.032383	0.031820	0.033098
MSE	0.001702	0.001642	0.001967
RMSE	0.041257	0.040523	0.044350
R2	0.979122	0.979858	0.975875

**Figure 12:**
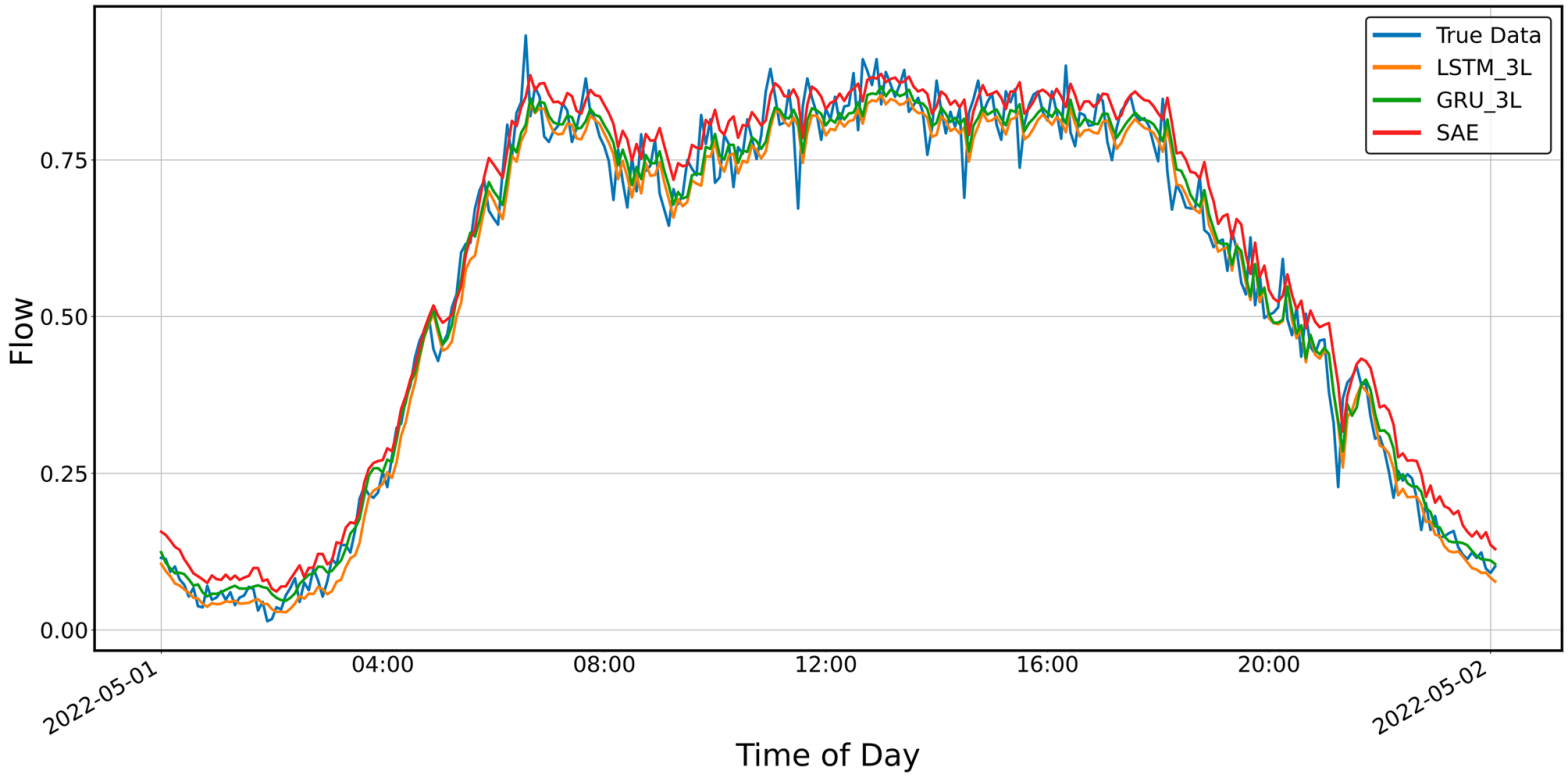
Prediction result for SAE and 3-layer LSTM and GRU models, prediction period 24 h.

**Figure 13:**
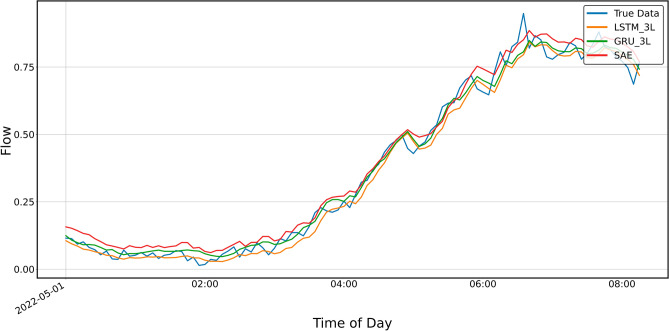
Prediction result for SAE and 3-layer LSTM and GRU models, prediction period of 8 h.

Figures 12 and 13 show the prediction results of the three-layer models for shorter periods, 24 h and 8 h, respectively. It can be seen from the graphs that the predictions do not keep up with the faster changes in the actual data. It is particularly clear in the upper, approximately flat part of the graph shown in Fig. 8 and the previous ones, i.e., Figs. 4, 5, 6 and 7.

**Figure 14:**
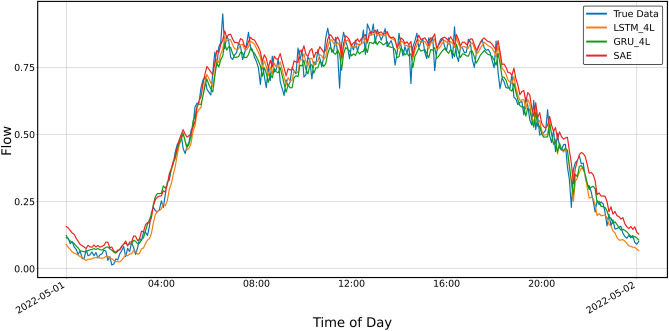
Prediction result for SAE and 4-layer LSTM and GRU models, prediction period 24 h.

**Figure 15:**
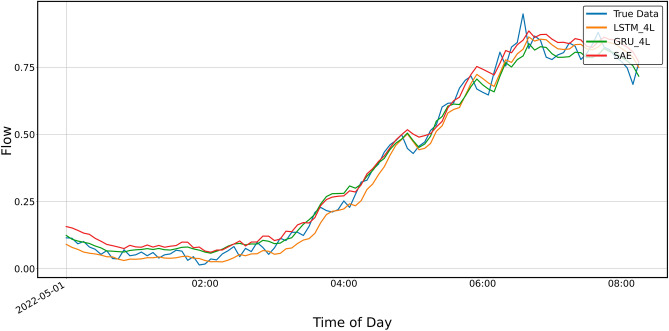
Prediction result for 4-layer LSTM and GRU models, prediction period of 8 h.

The prediction results of the SAE and four-layer LSTM I GRU models are shown in Figs. 14 and 15. As with the three-layer models, the predictions do not keep up with the rapid changes in real data.

## Discussion

Preparing models, especially LSTM, proved to be a relatively time-consuming task. Reduction of calculation time proved to be feasible when the graphics card computational resources were included in learning the models, which reduced the required time by about 400 times. This problem could become significant if the trained models were implemented in devices with relatively small computational resources, e.q. in intelligent traffic signs^19^. Furthermore, the software must be updated due to changing traffic conditions. From this point of view, the SAE model proved to be the best, i.e., the least energy-intensive model, followed by GRU in second place and LSTM in third position. The conclusions of publication^42^ regarding the significantly lower computational cost of the GRU model compared to LSTM were confirmed in this way. 

While computational needs may be necessary for specific applications, prediction accuracy is undoubtedly a more important criterion for evaluating model quality. Two observations should be mentioned at this point:

1. At the learning stage, the dependence of losses on the type of internal architecture of the models, that is, on the number of inner layers, is clear. It can be observed that with an increase in the number of layers, the accuracy of models (GRU and LSTM) increases, although this is not a linear relationship. As mentioned above, the best learning results were achieved by models containing an even number of layers, i.e., two or four. Among them, four-layer models showed the lowest learning errors. This effect can be observed in Figs. 4 and 5.
2. As seen in Fig. 6, the SAE model proved to be the least accurate of the tested models. The LSTM model, on the other hand, appears slightly more accurate than the GRU model with a similar architecture.

The resulting prediction accuracy is expressed by the metrics shown in Tables 1, 2, 3 and 4 as with the learning process, which depends on the architecture of the models. Single-layer LSTM and GRU models gave the best results (Table 1), which seems to contradict the above remarks on the learning process. On the other hand, LSTM I GRU models with an even number of inner layers gave significantly worse results than training with real data. Overall, the three-layer models proved to be the most accurate. The metrics shown in Table 3 are fairly even, but it can be seen that the GRU model performs least favorably in this comparison.

The trained models obtained in the experiments described above were made using the Keras library, allowing them to be saved in HDF5 binary format. They can be implemented in edge devices such as smart traffic signs. Suppose the target device has sufficient resources and environment (Linux, Tensorflow). In that case, copying the binary model into the new environment and loading it into memory using the appropriate function may be sufficient. For a Keras model stored as HDF5, the ‘keras.models.load_model (‘my_model.h5’)’ command can be used. In the case of limited resources or a different hardware platform, it will be necessary to convert the binary model to a format supported by the target device. This can be done using the TensorFlow Lite Converter tool, which can convert the TensorFlow and Keras models to the TensorFlow Lite (.tflite) format, designed to run on devices with limited computing resources and supported by different programming languages. An alternative solution is to use ONNX (Open Neural Network Exchange), an open standard for machine learning models supported by many libraries and platforms. The Keras2onnx library allows Keras models to be converted into ONNX format, which can then be loaded and run with the appropriate ONNX tool in various programming languages. 

The example process of model implementation:

1. *Model conversion to TensorFlow Lite* The first step is to convert the HDF5 model to TensorFlow Lite format (.tflite) using the TensorFlow Lite Converter tool. This can be done in Python using the appropriate TensorFlow functions.
  2. *Implementation of TensorFlow Lite Micro* TensorFlow provides a version of TensorFlow Lite Micro, which *is specifically designed* to run on microcontrollers. One needs to compile the library for your specific hardware platform to use TensorFlow Lite Micro on a microcontroller.
  3. *Loading the model onto the microcontroller* After compiling the TensorFlow Lite Micro library, one needs to configure the microcontroller to load the .tflite model. It may require converting the model into a byte array and saving it in a C++ header file, which *is then included* in the project.
  4. *Invoking inference* After loading the model, one needs to write code that feeds input data to the model and performs inference. For this, you use functions provided by the TensorFlow Lite Micro library.
  5. *Updates and maintenance* Once the model is implemented, regular updates and maintenance will be required. This may include re-training the model on new data, updating the intelligent road sign software, and monitoring model performance in real-time.

Extensive information on implementing trained models in embedded devices can be found at ^43–45^.

One should remember the computational needs of implemented models. As mentioned above, LSTM models are characterized by the greatest needs in this regard, hence its implementation in edge devices can be problematic. In such a case, the more suitable choice is undoubtedly the GRU model. An interesting variant could also be the minimal gated unit (MGU) models, which were not covered in our research.

## Conclusions

This paper compared traffic prediction results obtained using three machine learning models tested with two types of data: the first was real data provided by PeMS^17^, and the second came from traffic simulations conducted with the software simulator Vissim. The comparison was made by using the simulation results as a test set and re-generating predictions using the prepared machine learning models. The results obtained using data from the Vissim simulator do not differ (or differ slightly) from those obtained using real data. It leads to confirmation of the high reliability of the Vissim simulator. Still, on the other hand, it confirms the validity of the assumption that such a method, applicable in off-line mode, can be useful rather in situations where the acquisition of real data is difficult for various reasons. Meanwhile, machine learning models can operate in real-time mode. Furthermore, machine learning and simulations performed with specialized software are applicable for autonomous adaptive traffic control systems^19,46,47^.

### Supplementary Information

Supplementary Information.

## Data Availability

All data generated or analyzed during this study are included in this published article (and its Supplementary Information files).
