# Supplementary figures and images for: Road traffic can be predicted by machine learning equally effectively as by complex microscopic model
Source: Sci Rep. 2023 Sep 4;13:14523. doi: 10.1038/s41598-023-41902-y (PMC10477175; doi:10.1038/s41598-023-41902-y)

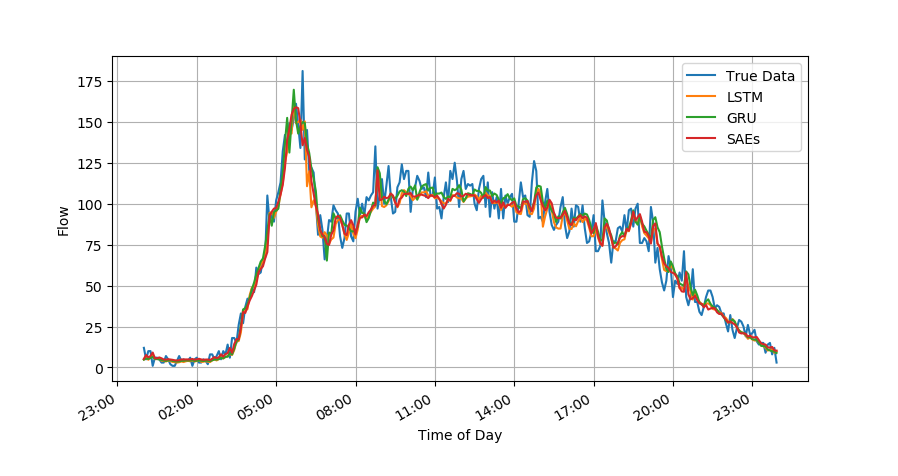

Supplement: Supplementary file 1 — Supplementary Information. [file 41598_2023_41902_MOESM1_ESM.zip › images/eva.png]

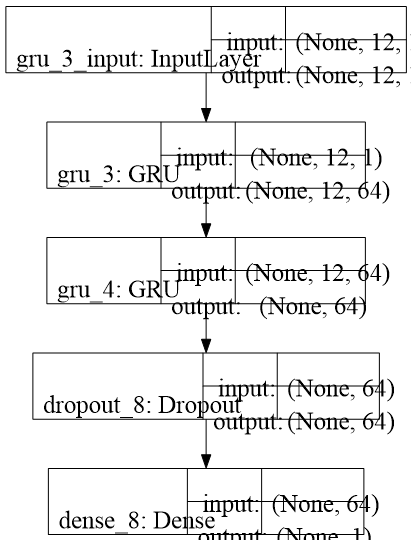

Supplement: Supplementary file 1 — Supplementary Information. [file 41598_2023_41902_MOESM1_ESM.zip › images/GRU.png]

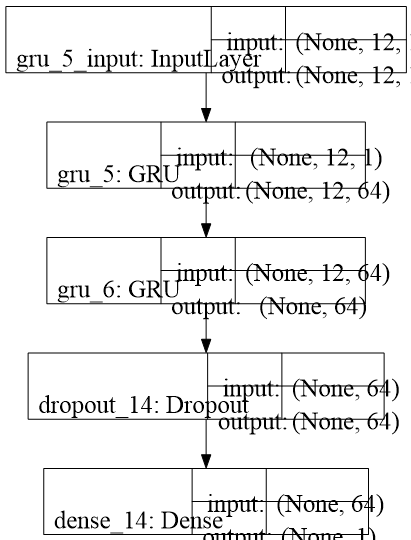

Supplement: Supplementary file 1 — Supplementary Information. [file 41598_2023_41902_MOESM1_ESM.zip › images/GRU_4L.png]

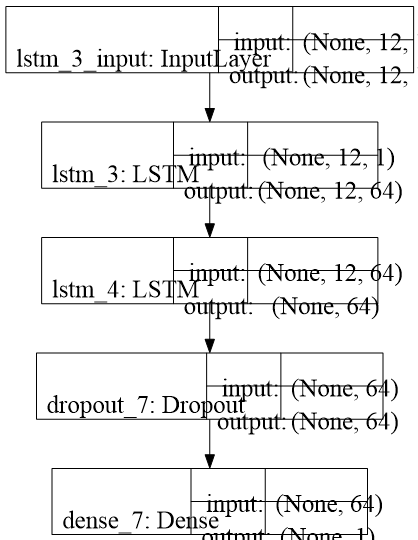

Supplement: Supplementary file 1 — Supplementary Information. [file 41598_2023_41902_MOESM1_ESM.zip › images/LSTM.png]

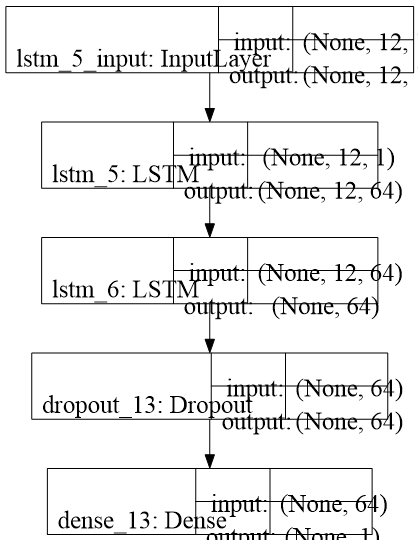

Supplement: Supplementary file 1 — Supplementary Information. [file 41598_2023_41902_MOESM1_ESM.zip › images/LSTM_4L.png]

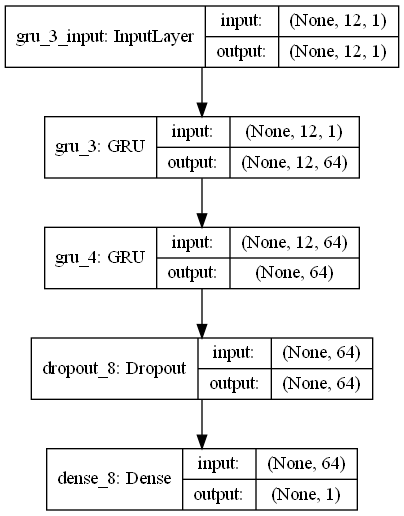

Supplement: Supplementary file 1 — Supplementary Information. [file 41598_2023_41902_MOESM1_ESM.zip › images/model_GRU.png]

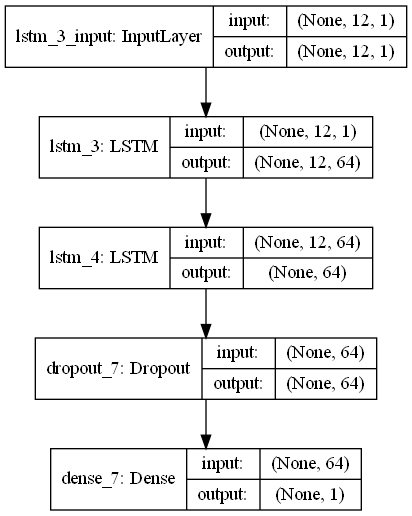

Supplement: Supplementary file 1 — Supplementary Information. [file 41598_2023_41902_MOESM1_ESM.zip › images/model_LSTM.png]

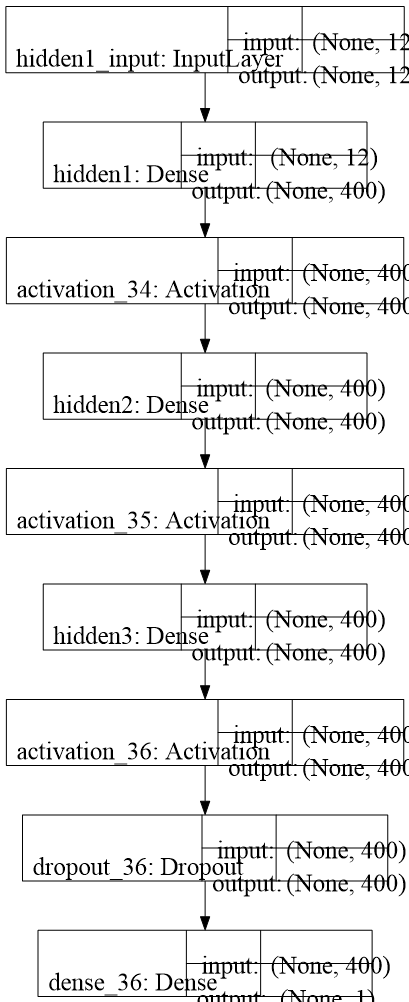

Supplement: Supplementary file 1 — Supplementary Information. [file 41598_2023_41902_MOESM1_ESM.zip › images/SAEs.png]
